# Influence of Donor Age, Donor Body Mass Index, and Harvesting Site on Cell Preparations from Human Adipose Tissue

**DOI:** 10.3390/ijms27031351

**Published:** 2026-01-29

**Authors:** Olga Hahn, Philipp-Kjell Ficht, Wendy Bergmann-Ewert, Juliane Meyer, Anne Wolff, Kirsten Peters

**Affiliations:** 1 Institute of Cell Biology, Rostock University Medical Center, 18057 Rostock, Germany; olga.hahn@med.uni-rostock.de (O.H.); philipp-kjell.ficht@med.uni-rostock.de (P.-K.F.); juliane.meyer@humanmed.com (J.M.); anne.wolff@med.uni-rostock.de (A.W.); 2 Core Facility for Cell Sorting and Cell Analysis, Rostock University Medical Center, 18057 Rostock, Germany; wendy.bergmann-ewert@med.uni-rostock.de

**Keywords:** stromal vascular fraction, mesenchymal stem/stromal cells, age, body mass index, harvesting site, therapeutic application

## Abstract

Adipose tissue includes various cell types beyond the typical adipocytes. The stromal vascular fraction (SVF) contains mesenchymal stem cells (MSCs), pericytes, and endothelial cells, which can be isolated from adipose tissue by mechanical and enzymatic methods. The composition of the SVF is heterogeneous, and donor factors such as sex, age, body mass index (BMI), and harvesting site are associated with variations in cellular composition and viability. The expression of specific surface markers, which determine the immunophenotype of the cells, can also vary. In this study, we investigated the effects of donor age, BMI, and harvesting site on cell yield, viability, and size. Our results showed that BMI significantly influenced cell yield and size, with overweight and obese donors yielding more cells than normal-weight donors. Additionally, cells isolated from the adipose tissue of the thighs/legs were larger than those from other areas. Flow cytometry showed considerable variability in SVF composition among donors. These results emphasize that SVF donor characteristics have a significant impact on cell yield, viability, and cell size, with the immunophenotype being highly donor-dependent. Understanding these factors is crucial for optimizing cell yield and defining populations for therapeutic applications of SVF cells.

## 1. Introduction

Adipose tissue plays a crucial role in storing energy as lipids, providing insulation to regulate body temperature, and acting as a cushion to protect organs from injury [1,2]. Additionally, adipose tissue serves as an active endocrine organ, releasing hormones and signaling molecules that regulate metabolism and inflammation [1]. The cellular composition of adipose tissue includes various cell types, with adipocytes being the most prominent. Since mesenchymal stem cells (MSCs) have also been detected in adipose tissue, this tissue has increasingly come to the fore as a cell source for regenerative therapies since the early 2000s [3]. By definition, MSCs are capable of self-renewal and proliferation and show little to no signs of cell differentiation, allowing them to differentiate into different cell types under certain conditions [4]. These properties make them a valuable tool in regenerative medicine, where they can support or completely replace damaged tissue such as cartilage, bone, and muscle. MSCs play a crucial role in tissue engineering by contributing to the formation of complex three-dimensional scaffolds of tissues and cells [5]. Adipose-derived MSCs (adMSCs) offer significant advantages over other MSC sources as they are more readily accessible and yield approximately 100 times more MSCs than bone marrow [6]. Furthermore, adMSCs share many characteristics with bone marrow-derived MSCs, including similar surface marker expression profiles [7,8]. These factors have established adipose tissue as a preferred source of MSCs for various tissue engineering applications and regenerative medicine research.

Technical dissociation of adipose tissue via mechanical and enzymatic processes yields a cell population known as the stromal vascular fraction (SVF) [9,10,11,12]. The SVF contains mesenchymal and hematopoietic stem cell types, as well as mature cells such as fibroblasts, smooth muscle cells, macrophages, lymphocytes, pericytes, and endothelial cells [8,13,14]. At the same time, adMSCs account for only <1% of the SVF [13,15]. The SVF and adMSCs demonstrate immunomodulatory effects by secreting growth factors and anti-inflammatory cytokines [15,16], promoting blood vessel formation, reducing inflammation, and thereby facilitating tissue repair [17]. Similarly, adMSCs suppress lymphocyte proliferation and inhibit dendritic cell activation, helping to regulate the immune response and promote healing [18]. However, studies indicate that the proportions of the various cell types in the SVF are heterogeneous, and donor characteristics, such as age and body mass index (BMI), are associated with variations in the cellular composition and viability of the SVF that may occur within the context of interindividual variability [19,20,21,22,23,24,25]. The extent of these variations differs depending on the parameter and may overlap with individual biological variability. Additionally, there are indications that the specific site of adipose tissue harvesting can influence the composition and characteristics of the SVF [21,22,26,27], and factors such as biological aging and overweight may affect SVF composition and the yield of the SVF and adMSCs [19,28,29].

Research suggests that the aforementioned donor characteristics may influence the expression of specific differentiation markers on the cell surface, which define their immunophenotype [24,30,31,32]. Consequently, it is essential to identify specific cell populations within the SVF based on characteristic surface markers delineated in prior studies [33]. Notably, CD34, a prominent marker for stem and progenitor cells, is frequently found in high amounts in the SVF, while CD45 indicates leukocytes, and CD31 and CD144 identify endothelial cells within the SVF [34,35,36,37]. One example of the complexity of analyzing the SVF is CD146, a well-known pericyte marker that is also expressed by endothelial cells [13,38]. Therefore, a single marker alone cannot identify specific SVF cell types; instead, combinations such as CD146+/CD31-/CD34-/CD144- are required to define pericytes [13]. Characterizing such surface marker constellations is crucial for understanding the functional roles of SVF cell subsets and, in turn, for optimizing adipose tissue-derived therapeutic applications.

The primary objective of this study was to investigate the impact of donor characteristics, including age, BMI, and harvesting site, on freshly isolated SVF and SVF-derived cells cultured for a maximum of 24 h. In addition, we analyzed a smaller dataset to gain initial insights into the relationship between these donor factors and SVF composition. By elucidating how these variables affect cell yield, viability, and composition, this research aims to support the development of more standardized and reliable protocols for the isolation, cultivation, and therapeutic use of adipose-derived cell populations.

## 2. Results

### 2.1. Characterization of the Donor Cohort

In this study, SVF and SVF-derived cells, specifically non-adherent, adherent, and adherent CD34-positive cells, were isolated and characterized from 120 human adipose tissue donations. Donor information was recorded alongside the tissue samples, with all personal data anonymized to protect the participants’ privacy.

Donor characteristics (sex, age, BMI, and harvesting site) were assessed at baseline to establish cut-off points and groupings for comparative statistical analysis, aimed to evaluate their impact on cell yield, viability, and cell size. In terms of sex distribution, 110 donors (i.e., 91.67%) were female, while 5 donors (4.17%) were male. No sex information was available for the remaining five donors (4.17%). Due to the significant imbalance in group sizes and the limited number of male donors, sex-specific effects were not explored further in the analysis.

**Figure 1:**
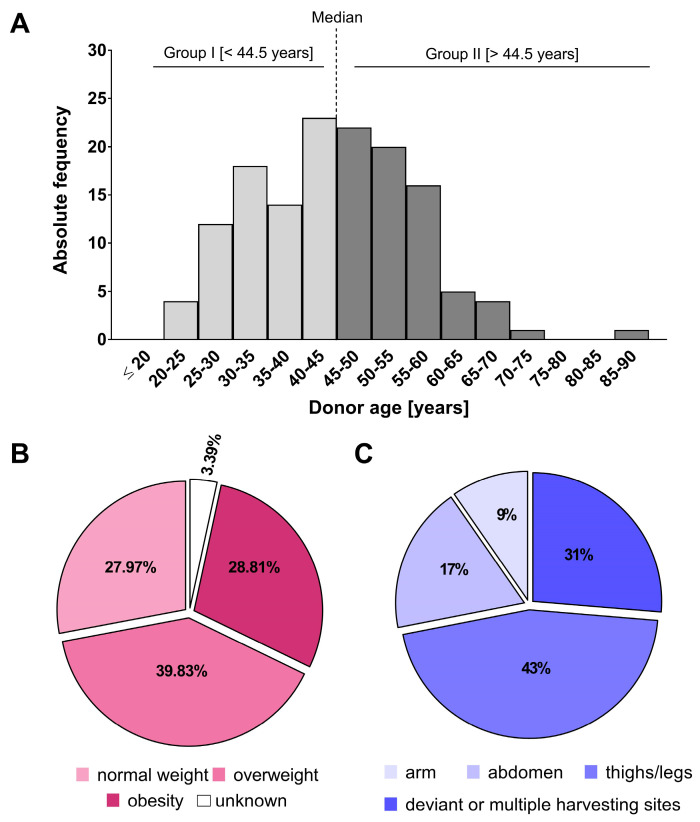
Characterization of the donor cohort. (**A**) Age distribution of the donors based on the median age of 44.5 years (group I < 44.5 years, group II > 44.5 years). (**B**) Relative BMI of donors according to clinical classification, and (**C**) relative proportions of anatomical origin of the harvesting sites (n = 114–120).

The distribution of the donor age (Figure 1A) revealed a non-Gaussian distribution (as assessed by the Shapiro–Wilk test) with a median age of 44.5 years. Therefore, the donors were divided into two groups based on the median age of 44.5 years: group I, donors with an age below the median age of 44.5 years (n = 60), and group II, donors with an age above the median age of 44.5 years (n = 60).

BMI was used to further characterize the donor cohort. Therefore, in the following analyses, the donors were divided according to the clinical classification for underweight, normal-weight, and overweight/obesity of the World Health Organization (WHO) and the National Institute of Health (NIH) (Table 1): group (I) normal-weight with a BMI between 18.5 and 25 (n = 33), group (II) overweight with a BMI between 25 and 30 (n = 47), and group (III) clinical obesity with a BMI above 30 (n = 34).

Four donors (3.39%) were excluded from further analyses because no personal weight and/or height information was available. Of the 118 donors, 27.97% were classified as normal-weight, meaning over 72% came from overweight or obese donors (Figure 1B). Specifically, 28.81% of donors were obese. None of the donors was classified as underweight. The size of the three groups included in the analysis ranged from 33 to 47 donors (approximately 27.97% to 39.83%), ensuring a relatively even distribution and providing a solid foundation for a robust statistical analysis of the effects of donor BMI on the available datasets.

In addition, the anatomical site of tissue harvest was investigated as a donation-specific parameter with potential influence: it was found that 52 donations (43%) came from the thigh/legs, followed by 21 donations (17%) from the abdominal area, and 11 (9%) from the arm (Figure 1C). The remaining 37 tissue donations (31%) were excluded from the analysis because (a) too few samples were taken from the harvesting sites, e.g., breast (n = 3), claves (n = 2), and hand (n = 1), or (b) tissue donations came from several harvesting sites of the same person that were later combined (31% with n = 30). To determine the influence of the anatomical area on cell yield, viability, or cell size, each harvesting site was compared with the others.

### 2.2. Determination of Cell Yield, Cell Viability, and Cell Size of the SVF and SVF-Derived Cells

The SVF was analyzed for cell yield, viability, and size at different time points; the first analysis was performed immediately after obtaining the SVF. Subsequent analyses were carried out within 24 h of cell cultivation. Since not all cells from the SVF could adhere to the culture surface, the cells in the supernatant, hereafter referred to as non-adherent (non-ad.), were also analyzed. In addition, the cell yield of all adherent cells (ad.) and adherent CD34-positive cells (ad. CD34+), as a subset of the adherent SVF, was quantified.

**Figure 2:**
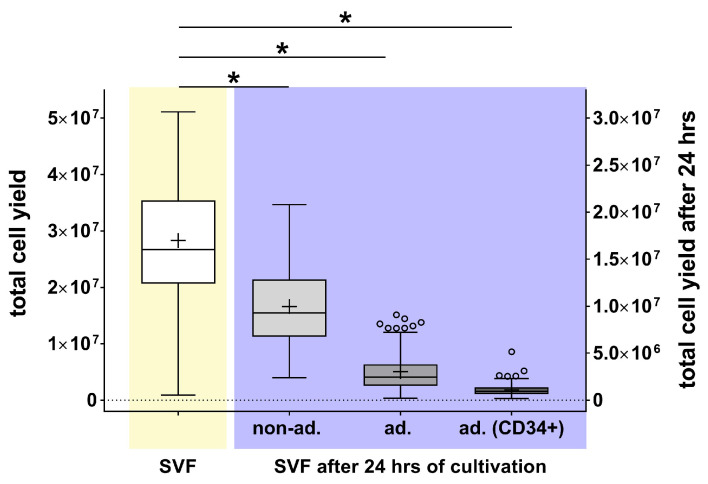
Quantification of the cell yield of SVF (yellow) and SVF-derived cells (purple). Cell yields of freshly isolated SVF and SVF-derived cells are presented as box plots, with medians, means (+), interquartile ranges, and minimum/maximum values as whiskers (Tukey biweight). Data points below or above the whiskers are defined as outliers (circles). The Shapiro–Wilk test indicated a non-Gaussian distribution; statistical significance was calculated using the Kruskal–Wallis test with Dunn’s multiple comparison post hoc test, * *p* < 0.05, significant compared to the SVF (n = 88–118).

The analyses revealed significant differences in the composition of the various SVF fractions (Figure 2). The SVF immediately analyzed after isolation contained an average of 2.67 × 10^7^ cells. After 24 h of cultivation, 34.68% (non-ad.: 9.26 × 10^6^) did not adhere to the culture surface, and 9.21% (ad.: 2.46 × 10^6^) adhered to the cell culture surface. Of these adherent cells, 37.76% (9.29 × 10^5^) were CD34-positive (ad. CD34+).

The cell viability of the SVF was between 76.99% immediately after isolation and 94.85% in adherent CD34-positive cells after 24 h of cultivation (Supplementary File, Figure S1). The relatively lower viability of the freshly isolated SVF can be attributed to the tissue-harvesting procedure and the subsequent transportation time of almost 24 h.

The cells of the SVF were, on average, about 9–10 µm in cell size, while the adherent cells of the SVF were about 12 µm in cell size after 24 h of cultivation (Supplementary File, Figure S1).

### 2.3. Impact of Donor Age

In the previous step, since the median age of the donors was 44.5 years, we divided the donor cohort into two groups: group I, comprising donors younger than 44.5 years, and group II, consisting of donors aged 44.5 years and older. In the next step, we examined how age influenced BMI in the age-disaggregated donor cohort and found no significant differences between groups I and II (mean BMI of group I was 28.19 vs. group II, 28.27; Table 2).

**Figure 3:**
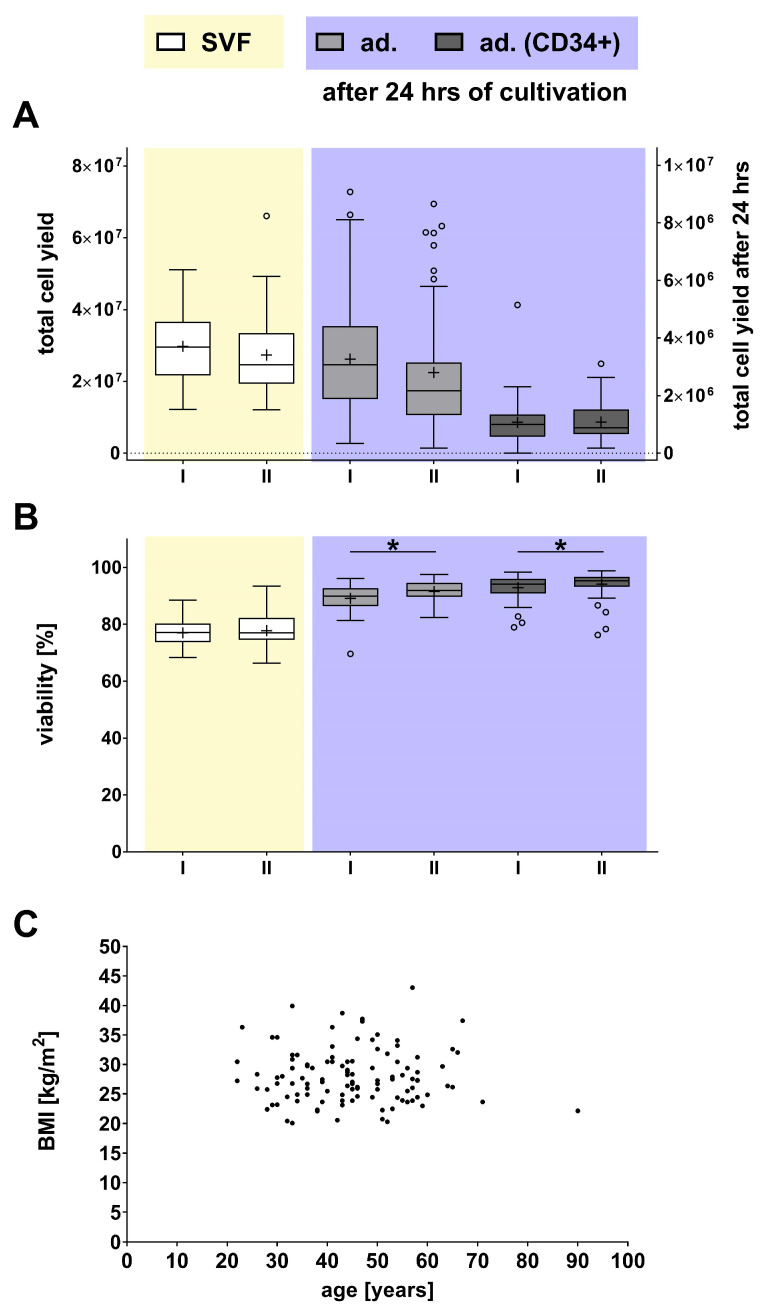
Comparative analysis of the effects of donor age on cell yield and viability of SVF (yellow) and SVF-derived cells (purple). (**A**) Cell yield and (**B**) cell viability of the freshly isolated SVF and SVF-derived cells are presented as box plots, with medians, means (+), interquartile ranges, and minimum/maximum values as whiskers (Tukey biweight). Data points below or above the whiskers are defined as outliers (circles). (**C**) Correlation analysis between age and BMI of the donors with Spearman correlation of −0.05 and a *p*-value of 0.58. Statistical significance was calculated using the Mann–Whitney test for datasets that indicated a non-Gaussian distribution (Shapiro–Wilk test). In contrast, datasets with a Gaussian distribution (Shapiro–Wilk test) were analyzed using Welch’s test, with a significance level of * *p* < 0.05 between group I (<44.5 years) and group II (>44.5 years, n = 108–115).

It should be noted that in none of the isolated cell populations (neither in the freshly isolated SVF nor in the SVF-derived adherent cell cultures) were statistical differences found in the number of cells obtained (Figure 3A).

However, we observed statistically significant differences in cell viability concerning the age of the donor (Figure 3B). While SVF viability was not affected by age, there was a slight but significant increase in the cell viability of adherent cells and adherent CD34-positive cells in group II (older donors) by 2.74% for adherent cells and 1.33% for CD34-positive cells. However, as the initial viability in these populations was already very high (at 91.05% and 94.85%), the relevance of this slight increase in viability must be considered carefully. Furthermore, a subsequent analysis revealed no correlation between the age and BMI of the donors (Figure 3C), leading to a more detailed investigation of the influence of BMI in the next step.

### 2.4. Effects of Donor BMI

As described above, BMI was used as a further criterion for classifying the donors into three groups: normal-weight (BMI < 25), overweight (25 < BMI < 30), and obese (BMI > 30). To assess the extent to which BMI influenced the cell yield, viability, and size, the previously measured data within these three groups were compared with each other in terms of their correlation. The characteristics of the three groups are summarized in Table 3.

**Figure 4:**
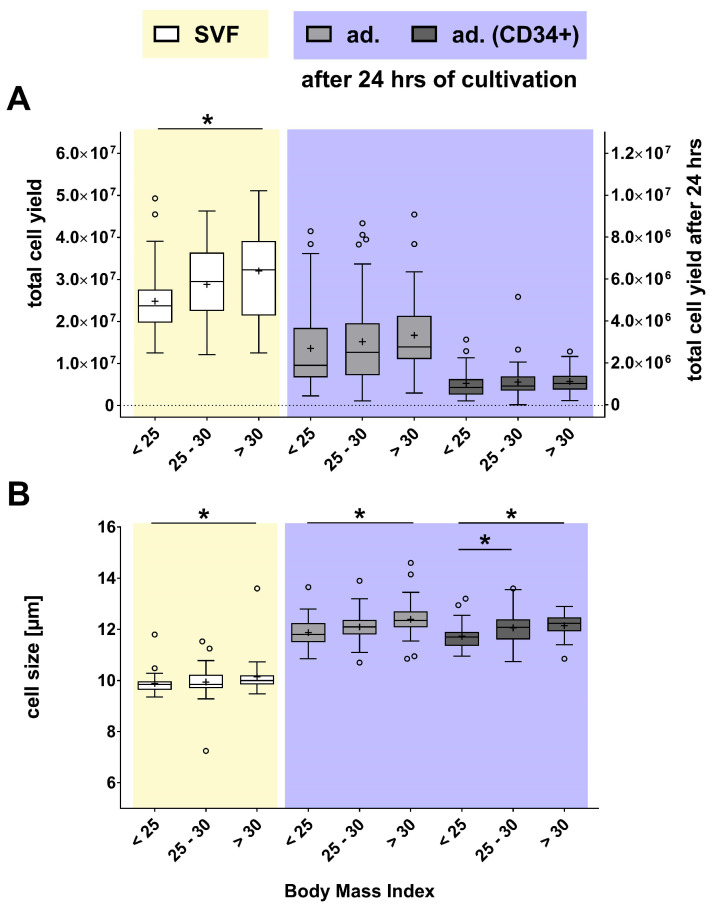
Comparative analysis of the effects of donor BMI on cell yield and size of SVF (yellow) and SVF-derived cells (purple). (**A**) Cell yield and (**B**) size of the SVF and SVF-derived cells are presented as box plots, with medians, means (+), interquartile ranges, and minimum/maximum values as whiskers (Tukey biweight). Data points below or above the whiskers are defined as outliers (circles). For datasets indicating a non-Gaussian distribution (Shapiro–Wilk test), statistical significance was calculated using the Kruskal–Wallis test with Dunn’s multiple comparison post hoc test. In contrast, datasets with a Gaussian distribution (Shapiro–Wilk test) were calculated using Ordinary One-Way ANOVA with Dunnett’s multiple comparison post hoc test, * *p* < 0.05 significance between normal-weight (BMI < 25), overweight (25 < BMI< 30), and obesity (BMI > 30); n = 106–113.

No significant differences were found between these three groups regarding donor age, ranging from 43 to 46 years (Table 3). However, it was found that a significantly higher cell yield (29.01% of cells) could be isolated from the SVF of obese donors with a BMI > 30 than from normal-weight donors with a BMI < 25 (Figure 4A). The SVF cell yield from overweight donors with a BMI between 25 and 30 was only 16.24% higher than that of normal-weight donors (not statistically significant). In contrast, and unexpectedly, the cell yield of non-adherent, adherent, and adherent CD34-positive cells did not differ between these three groups after 24 h of cultivation. BMI did not influence the cell viability of the cells (Supplementary File, Figure S2).

A higher BMI was associated with a larger cell size (Figure 4B). In the SVF of obese donors, a 2.71% increase in cell size was observed compared to normal-weight donors. A similar result was observed in adherent cells of the SVF after 24 h of cultivation: adherent cells from obese donors were 4.38% larger than those from normal-weight donors. The cell size of adherent CD34-positive cells was 2.81% larger in overweight and 3.50% larger in obese donors. Overall, the cell size of the SVF from donors with a higher BMI was larger than that of donors with normal-weight.

### 2.5. Cell Characteristics in Relationship to the Harvesting Site

The analysis of BMI and the harvesting site revealed no correlation (Spearman correlation of −0.2 and a *p*-value of 0.06). Given that previous research indicates that the specific harvesting site of adipose tissue can affect the SVF, a more detailed investigation of the harvesting site was undertaken as an additional analytical parameter. Therefore, donors were categorized based on the anatomical location from which the adipose tissue was harvested, and the potential effects of these sites on cell yield, viability, and size were assessed. Therefore, data from cells obtained from the thighs/legs, abdomen, and arms were compared. While the age of the donors was relatively similar across all groups (see Table 4), it is noteworthy that the BMI for donors from the arm liposuction tissue was higher, although not significantly, at 31.54, compared to the other groups.

**Figure 5:**
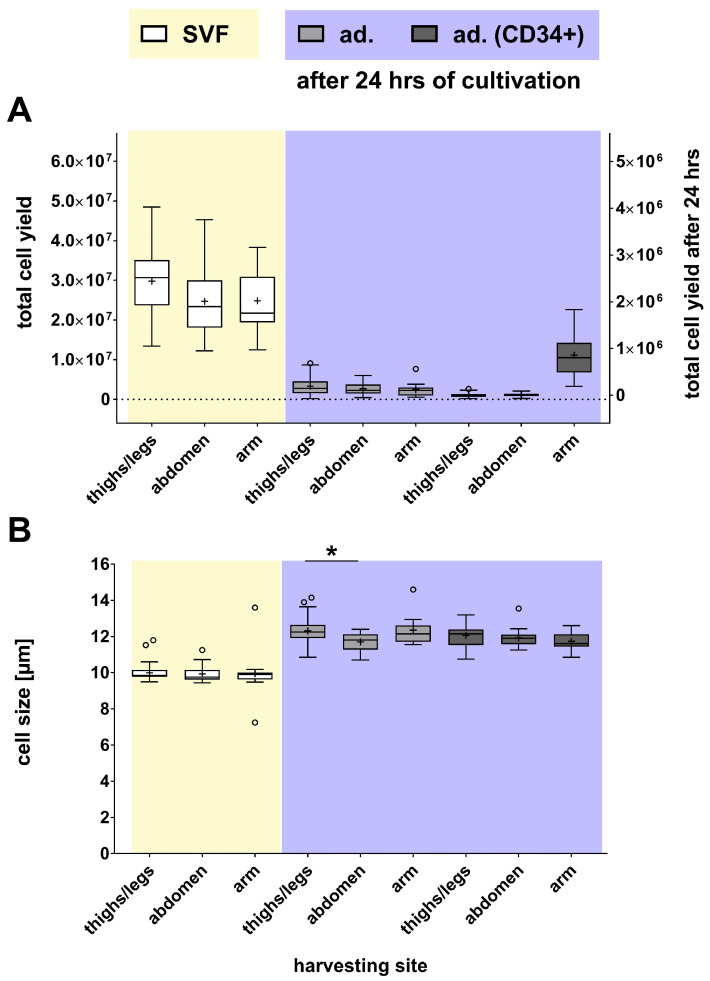
Comparative analysis of the effects of harvesting site on cell yield and size of SVF (yellow) and SVF-derived cells (purple). (**A**) Cell yield and (**B**) size of SVF and SVF-derived cells are presented as box plots, with medians, means (+), interquartile ranges, and minimum/maximum values as whiskers (Tukey biweight). Data points below or above the whiskers are defined as outliers (circles). For datasets indicating a non-Gaussian distribution (Shapiro–Wilk test), statistical significance was calculated using the Kruskal–Wallis test with Dunn’s multiple comparison post hoc test. In contrast, datasets with a Gaussian distribution (Shapiro–Wilk test) were calculated using Ordinary One-Way ANOVA with Dunnett’s multiple comparison post hoc test, * *p* < 0.05; n = 76–82.

There were no significant differences between these four groups in terms of cell yield (Figure 5A) and viability (Supplementary File, Figure S3). However, the harvesting site had an impact on cell size (Figure 5B). Specifically, the adherent cells originating from the thighs/legs were significantly larger than those from the abdomen (5.2%).

### 2.6. Flow Cytometric Characterization of Cell Populations Within the SVF

Following isolation, the SVF was characterized according to donor age and BMI classification, with a focus on cell populations and their relative proportions across 19 samples (Table 5).

**Figure 6:**
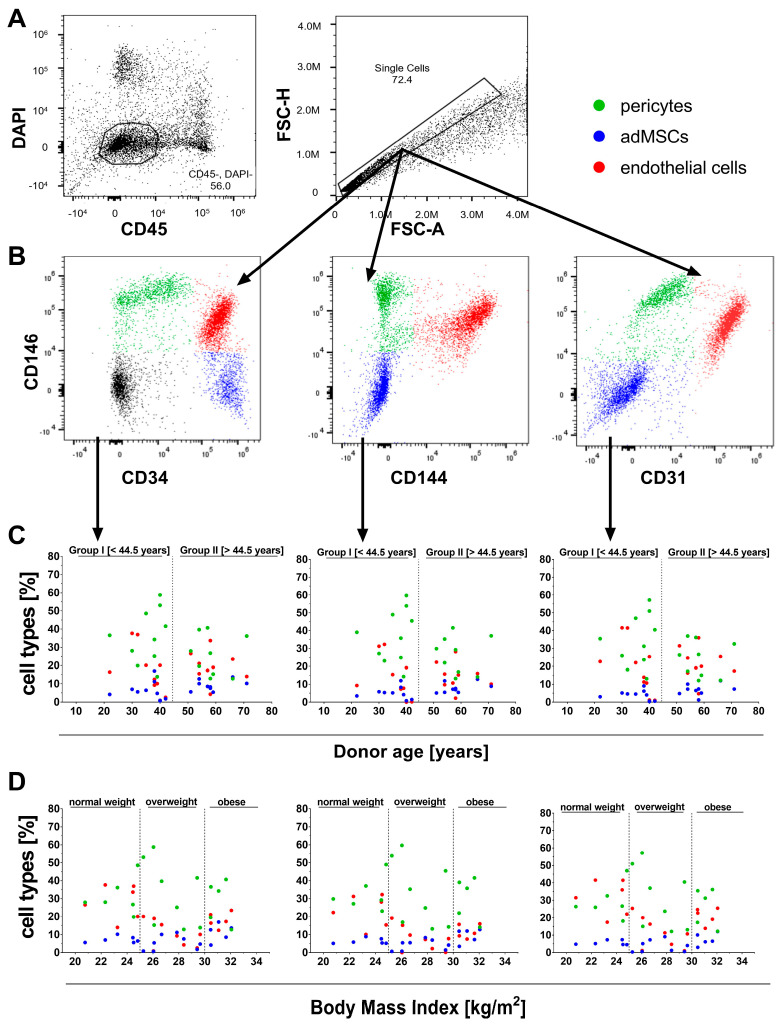
Characterization of cell types within the SVF. (**A**–**C**) Gating strategy for cell populations within the SVF. adMSCs, pericytes, and endothelial cells were discriminated in single viable CD45-negative cells (**A**) using dot plots of CD34, CD144, and CD31 versus CD146 (**B**). adMSCs identified as CD34+/CD31-/CD144-/CD146-, pericytes identified as CD146+/CD31-/CD34-/CD144-, and endothelial cells identified as CD31+/CD34+/CD144+/CD146+. Relative cell proportions within SVF were analyzed by donor age (**C**) and BMI (**D**).

Based on the literature, the characteristic surface marker profile of the SVF, including CD31, CD34, CD45, CD144, and CD146, was analyzed [13]. The following gating strategy was employed to determine cell identity and frequency: First, DAPI (4′,6-diamidino-2-phenylindole) was plotted against CD45 to select DAPI-negative (viable) and CD45-negative cells, thereby excluding hematopoietic cells (Figure 6A).

Doublet discrimination followed by plotting forward scatter area (FSC-Area) against forward scatter height (FSC-height). Subsequently, adMSCs, pericytes, and endothelial cells were then identified based on their respective marker combinations, such as CD34+/-, CD144+/-, and CD31+/- versus CD146+/- (Figure 6B). adMSCs were defined as CD34+/CD31-/CD144-/CD146-, pericytes as CD146+/CD31-/CD34-/CD144-, and endothelial cells as CD31+/CD34+/CD144+/CD146+. The proportions of these cell types were evaluated in relation to donor age and BMI classification, revealing no correlation with age (Figure 6C) or BMI (Figure 6D). Irrespective of the plotting strategy used (CD34 vs. CD146, CD144 vs. CD146, or CD31 vs. CD146, Figure 6C,D, and Table 6), the SVF contained 7–9% adMSCs, 24–27% pericytes, and 14–22% endothelial cells. The coefficients of variation were 33–45% for adMSCs, 39–44% for pericytes, and 42–52% for endothelial cells, underscoring interindividual variability in SVF-derived populations (Table 6).

### 2.7. Quantification of Cytokines in the Cell Culture Supernatant

Cell culture supernatants from the SVF were analyzed after 24 h of cultivation (n = 6). Cytokine profiles were examined relative to donor age and BMI classification. As shown in Table 7, the BMI values were comparable within groups I and II, and donor ages were also similar within each BMI category (normal-weight and overweight).

**Figure 7:**
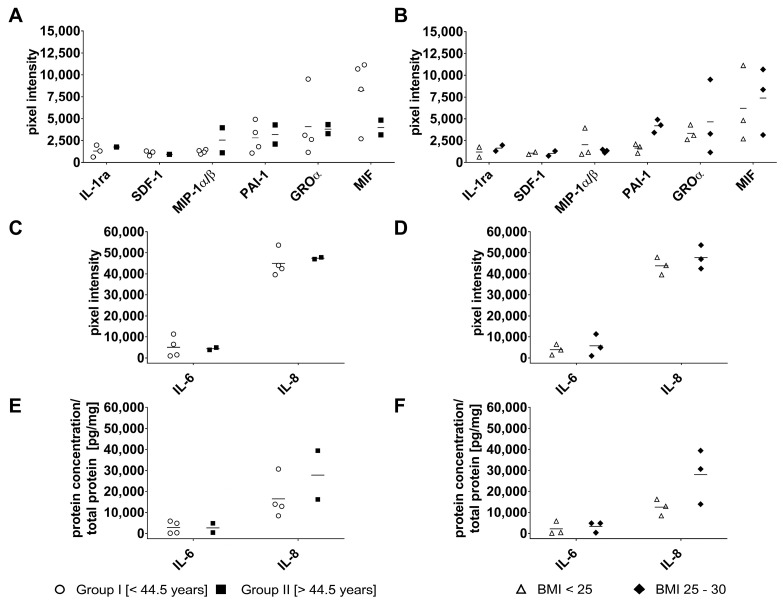
Cytokine secretion from the SVF in relation to donor age (**A**,**C**) and BMI (**B**,**D**). IL-6 (**E**) and -8 (**F**) concentration in SVF supernatants measured by ELISA. Data shown as individual values donor with mean (n = 6).

Of the 36 analytes examined, 8 cytokines—Interleukin (IL)-1ra, stromal cell-derived factor (SDF)-1, macrophage inflammatory protein (MIP)-1α/β, plasminogen activator inhibitor (PAI)-1, growth-regulated oncogene (GRO)α, macrophage migration inhibitory factor (MIF), IL-6, and IL-8—were detected with substantial but variable signal intensities (Figure 7A–D).

Cytokine secretion showed no significant association with donor age (Figure 7A,C) or BMI (Figure 7B,D), except for PAI-1, which was higher in donors with an elevated BMI compared to those with normal-weight donors.

Since IL-6 and IL-8 showed strong signals in the array analysis (Figure 7C,D), their concentrations were further quantified by ELISA. IL-6 secretion did not vary significantly with donor age or BMI (Figure 7E), whereas IL-8 levels tended to increase with both increasing age and elevated BMI (Figure 7F).

## 3. Discussion

Adipose tissue is widely recognized as an abundant and readily accessible source of stem cells for regenerative medicine, with SVF considered advantageous due to its high yield and minimal donor morbidity [39,40]. Both the SVF and adMSCs possess significant regenerative potential [7,8,15], which is thought to occur primarily through paracrine signaling, rather than direct cell differentiation [7,30,41]. The heterogeneous cellular composition of the SVF is thought to further enhance these effects by creating a supportive signaling environment that promotes tissue repair and regeneration [14,41]. As donor-related factors, such as age, BMI, and harvesting site, can influence the SVF yield and properties, a detailed characterization is essential to ensure therapeutic outcomes [19,20,22,24,25].

### 3.1. Donor Cohort

In 2023, female patients accounted for 84.85% of all liposuction procedures worldwide, while male patients accounted for 15.15% [42]. In our cohort, 92% of liposuctions were performed on female donors, slightly above the global average, likely reflecting regional differences [43]. According to the International Society for Aesthetic Plastic Surgery, 43.1% of donors who underwent liposuction in 2023 were between 35 and 50 years old (average age of 43 years) [43]; thus, the median age of 44.5 years in our cohort closely matches the global trend. Our donor cohort was predominantly overweight or obese, with the thighs/legs and abdomen as the most frequent harvesting sites. This cohort represents a distinct subgroup of the general population, characterized by financial means (as liposuction is often self-funded) and appearance orientation. The age distribution indicates that female donors in group I remained largely premenopausal, whereas donors in group II (>44.5 years) were predominantly peri- or postmenopausal, potentially affecting adipose tissue characteristics and metabolic activity.

### 3.2. Correlation Between Donor Age, Donor BMI, Harvesting Site, and the Characteristics of Freshly Isolated SVF and SVF-Derived Cells

Age might significantly influence cell quality by accumulating molecular damage and promoting senescence [44,45]. In this study, no significant differences in cell yield were observed between age groups (group I < 44.5 years; group II > 44.5 years), contrasting with studies reporting age-related effects on yield, although age-dependent variations in differentiation/migration potential exist. However, other studies have found significantly lower SVF yield in older donors (45–75 years) compared to middle-aged donors (38–44 years) [24,32,46]. The basis for these discrepancies remains unclear but may involve donor selection criteria, tissue quality, and protocol variations [47,48,49]. Cell viability across studies shows excellent rates for SVF cells (94–95%) and adMSCs (93.12–96.14%) [50], confirming our findings, although reduced viability in older donors has been reported [51]. Regardless of age-related variations, the viability of the isolated cells generally remains high.

We also investigated BMI effects by categorizing the donor cohort into normal-weight (BMI < 25), overweight (25 < BMI < 30), and obese (BMI > 30) donors. Overweight and obese donors showed an increased SVF cell yield immediately after isolation, likely reflecting the higher adipose tissue volume. In contrast, no significant differences were found in the SVF and the SVF-derived cells. These results align with prior research reporting higher SVF yields in individuals with a BMI above 25 [19], although another study found no effect of BMI [52]. Higher SVF cell yields in individuals with a higher BMI may partly result from increased CD45+ immune cell infiltration associated with obesity-related low-grade inflammation [28,29,53,54]. For example, a higher proportion of neutrophils (a subset of CD45+ cells) has been observed in the visceral adipose tissue of obese individuals, which correlates with increased insulin resistance and systemic inflammation [54,55]. Obesity-related inflammation is often associated with increased infiltration of pro-inflammatory immune cells (such as Th1 cells and macrophages) and consequently contributes to the overall CD45+ cell population in the SVF [56]. BMI did not affect cell viability in this study, contrasting with Karadag et al., who observed higher cell viability in donors with higher BMI [24]. This discrepancy may reflect their smaller cohort (n = 30) and the use of mean BMI without sub-classifications, whereas our study employed BMI-stratified analyses [24]. Notably, our study also found cell sizes within the SVF from donors with higher BMI. The obesity-associated inflammatory milieu and adipocyte hypertrophy are known to increase the size of CD34+ adMSCs [28,29,57,58,59,60,61], likely contributing to this observation. Furthermore, adipose stem cells may adapt to chronic inflammation through organelle development, promoting cellular enlargement [28,62].

The optimal method of adipose tissue harvesting for stem cell isolation remains unclear due to contradictory findings in the literature. Tsekouras et al. reported higher SVF and adMSC yields from femoral adipose tissue [50], while Jurgens et al. found no significant differences across five different harvesting sites [21]. In our study, the harvesting site had no evident influence on cell yield or viability, but thigh/leg tissue yielded significantly larger cells, possibly due to chronic pro-inflammatory activation [28,29,57,58]. The BMI of donors from whom adipose tissue was harvested from the arm was significantly higher than that of donors from other sites. Women typically store more subcutaneous adipose tissue in the gluteal–femoral region [6,63].

The relationship between donor characteristics (age, donor BMI, harvesting site) and SVF/adMSC properties is complex. BMI primarily affects cell yield (higher BMI → higher cell yield) with minimal viability effects, while also influencing cell size and potentially differentiation, immunological, and angiogenic properties [64]. In contrast, age and harvesting site showed no significant effects on cell yield, viability, or cell size, suggesting these parameters may be less critical for donor selection. Future studies should clarify how donor factors affect SVF composition, cellular functionality, and therapeutic efficacy to optimize donor selection and develop predictive models.

### 3.3. Immunophenotype of SVF Cell Populations

Specific cell populations are defined by distinct surface marker patterns [33]. This study analyzed CD34, CD45, CD31, CD144, and CD146 to distinguish three key populations within the isolated SVF: adMSC, pericytes, and endothelial cells. Pericytes dominated (24–27%), exceeding International Federation for Adipose Therapeutics (IFATS) and the International Society for Cellular Therapy (ISCT) expectations of 3–5%, while endothelial cells (14–22%) aligned with the prediction of 10–20% [13]. However, adMSCs were underrepresented (7–9% versus 15–30%). These deviations likely reflect our modest sample size (n = 19) and the challenging distinction between pericytes/adMSCs, which share a mesenchymal origin, differentiation potential, and dynamic CD34 expression that declines rapidly in cell culture [65,66] and encompasses both endothelial/perivascular subsets and variable gating criteria across studies [13,65,67].

Previous research suggests that donor weight has a notable impact on the immunophenotype of adMSCs [64]. Obese donors appear to exhibit higher expression of specific markers, such as CD146, which is associated with the presence of higher amounts of pericytes and thus increased adipogenic differentiation capacity. In contrast, overweight donors show lower CD146 expression and indications for a higher angiogenic potential [64]. In our study, overweight donors showed higher variability in pericyte populations, consistent with this heterogeneity in stromal cell subsets influenced by BMI. The inflammatory environment associated with obesity may further alter the characteristics of adMSCs during in vitro culture, affecting their immunologic profile and differentiation capacity [64].

Contrary to reports of age-related decline in adMSCs, our study found no significant correlation with donor age or BMI [20,51,68,69]. solation techniques (enzymatic/mechanical) and interindividual variation contribute to these discrepancies. Larger cohorts with standardized flow cytometry are essential to resolve donor effects on SVF composition and therapeutic relevance [34,37,48,49].

### 3.4. Cytokine Secretion Profile

Obesity is associated with pro-inflammatory SVF cytokine profile, including elevated TNF-α, IL-6, and chemokines [70,71,72,73]. Disease-specific analyses typically show only subset differences [74]. Our SVF culture supernatants exhibited limited secretion (8 of 36 analytes detectable), reflecting heterogeneous composition and modest basal activity.

PAI-1 was the primary BMI-sensitive cytokine, with higher levels in overweight donors—consistent with SVF-derived stromal cells as primary PAI-1 sources—contributing to insulin resistance and thrombosis [56,75,76,77,78,79,80,81,82]. Multiple studies report higher basal IL-6 secretion from the SVF of obese donors, reflecting increased leukocyte/macrophage content [83,84,85,86,87]. However, our study found no IL-6 differences by age or BMI, despite robust array signals. This may reflect our narrow BMI range, small cohort size (n = 6), and culture conditions that attenuate inflammatory priming, highlighting SVF’s context-dependent responsiveness [74,88]. By contrast, IL-8 levels increased with both age and BMI, consistent with its association with visceral adiposity, insulin resistance, and metabolic inflammation [73,89,90]. Although age-related IL-8 elevation in the SVF has been associated with stromal senescence and metabolic stress [91,92,93,94,95], our study showed no overall age-dependent differences in the cytokine profile.

Together, PAI-1 and IL-8 emerge as key BMI-responsive SVF cytokines with potential implications for regenerative capacity and donor selection [96,97]. The limited cytokine panel and donor heterogeneity underscore the need for larger studies with broader BMI ranges.

### 3.5. Study Limitations

This study identified correlations between donor characteristics and SVF properties. In this autologous clinical context, donor variation is inherent; observed group differences are thus likely to exceed technical noise. However, several limitations affect their interpretation and clinical relevance. The cohort was predominantly female (~92%) and excluded lipedema patients, reflecting typical cosmetic surgery demographics. This bias precludes sex-specific analyses despite known adipose tissue dimorphism, as thigh-derived cells yield higher numbers due to women’s gluteofemoral fat preference [6,22,26,63]. Additionally, SVF assessments capture only 24h snapshots of basal activity and lack long-term functionality. Stratification was limited by unknown hormone/menopausal status, medications, and comorbidities affecting liposuction efficiency [24,98,99]. Chronological age and BMI serve as imperfect proxies for biological aging; DNA methylation or telomere length would provide greater precision [100,101]. Enzymatic digestion remains the preferred standard for cell viability, yet it introduces batch variability and regulatory complexity [8,49,102,103]. Automated closed systems improve standardization but lack full clinical validation [104,105]. Clinically, the SVF from obese donors may require distinct priming strategies, while long-term engraftment and patient heterogeneity remain uncharacterized [105].

In summary, this study advances understanding of donor-driven SVF variability but underscores technological, biological, regulatory, and economic barriers to translation.

## 4. Materials and Methods

### 4.1. Isolation of the SVF

Adipose tissue was obtained through tumescence-based liposuction over a period from May 2020 to May 2022. The aspirated tissue was transported to the processing site at room temperature overnight and processed after 24 h. Primary human adMSCs were isolated from the liposuction tissue of 120 healthy donors using a previously described standardized and established protocol [106]. For this purpose, 30 milliliters of lipoaspirate was digested with 0.15 units/mL collagenase (NB4 from Clostridium histolyticum; Nordmark, Biochemicals, Uetersen, Germany) in PBS with Ca^2+^ and Mg^2+^ (Cytiva Hyclone™, Fisher Scientific, Schwerte, Germany) for 30 minutes at 37 °C with constant shaking. By using a specific enzyme activity of 0.15 U/mL during digestion, possible batch deviations of the collagenase were avoided. Subsequently, repeated filtration steps using cell strainers (100 µm and 40 µm) were performed, followed by washing with 10 mL PBS containing 10% FBS, sedimentation, and centrifugation steps (10 and 5 min, 400× *g*, RT). After discarding the supernatant, the so-called stromal vascular fraction was resuspended in 12.5 mL cell culture medium (Dulbecco’s Modified Eagle Medium (DMEM)) with high glucose (4.5 g/L) containing 1% penicillin/streptomycin (penicillin: 100 U/mL; streptomycin: 100 mg/mL, Thermo Fisher Scientific, Schwerte, Germany), 10% FBS and 0.4% GlutaMAX™. To determine the cell yield, viability, and cell size, 500 µL of the cell suspension was used. The remaining SVF was seeded onto a 75 cm^2^ TCPS cell culture flask and incubated for 24 h at 37 °C and 5% CO_2_ in a humidified atmosphere.

### 4.2. Determination of Cell Yield, Cell Viability, and Cell Size

To determine and analyze the cell yield, viability, and size of the SVF after isolation, the NucleoCounter^®^ NC-3000TM (Chemometec, Allerod, Denmark) ‘Viability and Cell Count Assay’ was used. The measurements were performed according to the manufacturer’s instructions using volume-calibrated cassettes (Via1-Cassette™, Chemometec, Allerod, Denmark) containing two immobilized fluorophores: acridine orange (staining all cells) and 4′,6-diamidino-2-phenylindol (staining dead cells) (both from Chemometec, Allerod, Denmark). Measurements of the sample were performed twice to increase the accuracy of the calculated cell numbers, viabilities, and cell sizes.

### 4.3. Separation of Adherent and Adherent CD34-Positive Cells from the SVF

Twenty-four hours after isolation, the CD34-positive cells were isolated using the Dynal^®^ CD34 precursor cell isolation system (Invitrogen, Karlsruhe, Germany), as previously published [107]. For this purpose, the non-adherent cells were collected and centrifuged at 500× *g* for 5 min at room temperature to remove erythrocytes and cell debris. Afterwards, the supernatant was filtered over a 40 µm cell strainer and stored at −80 °C for further cytokine analysis (Section 4.5 and Section 4.6). The remaining cell cultures with the adherent cells were washed twice with PBS. The cells were then incubated for 5 min. at 37 °C and 5% CO_2_ in a humidified atmosphere with CD34 antibody-coupled magnetic particles in cell culture medium. Afterward, the non-adherent beads were removed by washing them twice with PBS. The cells were then detached enzymatically with trypsin, and the adherent cells were counted. Repeated steps of magnet exposure and washing with PBS/10% FBS on a rotating mixer at 4 °C purified the CD34-positive cell suspension. During the separation procedure, 200 µL of the non-adherent, adherent, and CD34-positive cells were used to determine cell yield, viability, and size, as described in Section 4.2. Lastly, the CD34-positive cells were suspended in a cell culture medium for further analysis.

### 4.4. Analysis of Surface Marker Expression of the SVF

In addition, a smaller group (n = 19 with a mean age of 46.53 ± 13.26 years and a BMI of 27.11 ± 3.33 kg/m^2^), randomly selected (6 normal-weight, 8 overweight, and 5 obese), was analyzed by spectral flow cytometry to characterize the composition of the cell type within the SVF. For this purpose, the characteristic pattern of surface marker expression was determined, and the individuals were compared with one another to investigate interindividual variability. The following three cell populations within the SVF were analyzed in detail: pericytes, adMSCs, and endothelial cells (progenitor cells).

For analysis of the composition of the SVF, 1 × 10^6^ total cells were filled up with autoMACS running buffer (Miltenyi Biotech, Bergisch Gladbach, Germany) to 5 mL and centrifuged for 5 min at RT at 400× *g*. The cells were incubated with blocking buffer (Human Tandem Signal Enhancer, Miltenyi Biotech) for 10 min. on ice to prevent nonspecific binding of fluorophores, followed by an additional 20 min. incubation with CD31-BV605 (1:20, BioLegend, San Diego, CA, USA), CD34-PE (1:50, BioLegend), CD45-FITC (1:50, Miltenyi), CD90-APC (1:20, Biozol, Eching, Germany), CD144-PE-Vio700 (1:20, Miltenyi), and CD146-BV711 (1:20, BioLegend). Subsequently, incubation was performed for 20 min on ice, followed by lysis of the erythrocytes with NH_4_Cl for 10 min. on ice. Prior to measurement, DAPI was added to discriminate between dead and live cells. Unstained SVF approaches were used to subtract autofluorescence. FMO controls were used for correct gate setting, and isotypes were used to control for unspecific Ig binding to Fc receptors.

### 4.5. Measurement of Cytokine, Chemokine, and Acute Phase Protein Levels Within SVF Cells

**Figure A1:**
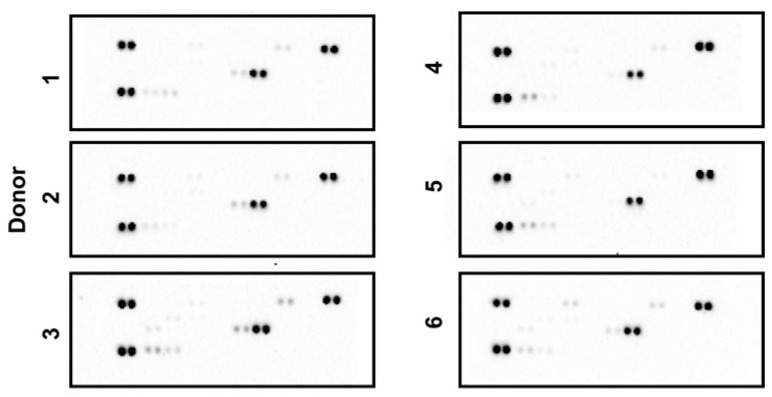
Membrane blots of the analysis of human cytokines, chemokines, and acute-phase proteins in the SVF of 6 donors (1–6). Proteins were detected using the ‘Proteome Profiler Human Cytokine Array Kit’ (pixel intensity of the protein spots) and quantified via densitometry analysis using QuickSpots software, Version 25.6.4.2. Spots on the membrane in duplicate represent each analyzed protein; n = 6.

The previously processed cell supernatants were used to detect 36 different cytokines, chemokines, and acute-phase proteins (Table A1) using a Proteome Profiler Human Cytokine Array Kit (R&D Systems, Minneapolis, MN, USA). To compare the results of individual donors, the total amount of protein was first determined using a DC protein assay kit (BIO-RAD Laboratories GmbH, Munich, Germany), following the manufacturer’s instructions. Subsequently, 300 µg protein/donor was used for the cytokine detection using the kit according to the manufacturer’s instructions. For this, chemiluminescence blots were recorded using ChemiDoc XRS (Bio-Rad Laboratories GmbH, Munich, Germany; Figure A1), and the protein intensity was quantified by densitometric analysis of the blot with QuickSpots software Version 25.6.4.2 (Ideal Eyes Systems).

### 4.6. Measurement of the Total Protein Amount

The determination of total protein concentration was performed using 5 µL of supernatant collected and stored as mentioned above, with a DC protein assay kit (BIO-RAD Laboratories GmbH, Munich, Germany) according to the manufacturer’s instructions. The fluorescence was measured using a microplate reader (TECAN) at 650 nm. For the calibration curve, BSA standard concentrations of 1.44, 1, 0.5, 0.25, 0.125, and 0 mg/mL were prepared by serial dilution. The samples and the standards were measured in a microplate reader (TECAN) at a wavelength of 650 nm.

### 4.7. Quantification of IL-6 and IL-8 by ELISA

Based on the proteome profiler results, the concentrations of the two highest relative cytokine/chemokine values, in this case IL-6 and IL-8, were then determined by ELISA (R&D Systems, Inc., Minneapolis, MN, USA) according to the manufacturer’s instructions. Absorbance was measured using a microplate reader (TECAN) at 450 nm and a reference wavelength of 540 nm. The concentrations of IL-6 and IL-8 were measured in picograms per milliliter (pg/mL) and normalized to the total protein amount.

### 4.8. Data Processing

Cell yields, viabilities, and sizes were determined in duplicates to increase accuracy. Therefore, the corresponding mean values were calculated for each measurement. The cell concentrations were multiplied by the total volume to obtain an absolute number for comparison with data from the different isolation points. The mean values for cell yield, viability, and size were then combined with the donor data to create a data matrix for statistical analysis.

For further characterization of the donor cohort, BMI was calculated as the ratio of body weight (in kg) to the square of body height (in m), using the following formula

$$MI\;\left[\frac{kg}{m^{2}}\right]=\frac{Body\;Mass\;\left[kg\right]}{{Body\;Height}^{2}\;\left[m^{2}\right]}$$

### 4.9. Statistical Analysis

All measurements were performed independently on the SVF immediately after isolation and on the SVF-derived cells after 24 h of cultivation from 120 donors, each in duplicate. The mean of each duplicate was used for one individual. Data were visualized and statistically analyzed with Microsoft Excel 2010 and GraphPad Prism, Version 7 (GraphPad Software Inc., San Diego, CA, USA). The data are presented as boxplots. The horizontal line within the box plot indicates the median, and the mean (+) is also shown, with whiskers extending to the minimum and maximum data points. For datasets indicating a non-Gaussian distribution (Shapiro–Wilk test), statistical significance was calculated using the Kruskal–Wallis test with Dunn’s multiple-comparison post hoc test or the Mann–Whitney test. In contrast, datasets with a Gaussian distribution (Shapiro–Wilk test) were calculated using Welch’s test or Ordinary One-Way ANOVA with Dunnett’s multiple comparison post hoc test. The significance level was set to a *p* value of 0.05.

Flow cytometry data were analyzed to quantify cell populations, as described in Section 4.4, using FlowJo software (version 10.10.0). Dot plot proportions of eight individuals were used to calculate mean values and standard deviations. The coefficient of variation was calculated by dividing the standard deviation by the corresponding mean to estimate interindividual variability of the SVF composition using the following equation

$$\mathrm{Coefficient}\;\mathrm{of}\;\mathrm{variation}\;V=\frac{standard\;deviation}{mean}=\frac{s}{\bar{x}}$$

## 5. Conclusions

This study, which is based predominantly on female liposuction, demonstrated that donor characteristics significantly influence SVF properties.

(I) Donor age slightly increased cell viability (+2.74% in adherent cells, +1.33% in CD34-positive cells for the >44.5 years cohort) without affecting cell yield or size.
(II) BMI (body weight/height^2^) significantly increased both cell yield and size in overweight/obese donors, while viability remained unaffected.
(III) Harvesting site influenced cell size, with thighs/legs yielding the largest cells, but showed no effects on cell yield or viability.
(IV) Flow cytometry revealed high interindividual variation in SVF composition.
(V) Cytokine secretion exhibited a tissue-specific profile (8/36 analytes detectable), with PAI-1 responding primarily to BMI and IL-8 increasing with both age and BMI.

These donor-specific effects go beyond quantitative measurements and may potentially affect functional properties such as proliferation and differentiation. Comprehensive donor profiling is therefore essential for optimizing personalized regenerative therapies.

## Figures and Tables

**Table 1:** Classification of donor BMI according to the international clinical grading.

Category	BMI [kg/m^2^]	Absolute Frequency	Relative Frequency [%]
underweight	<16–18.5	0	0
normal-weight	18.5–25	33	27.97
overweight	25–30	47	39.83
obesity	>30	34	28.81

**Table 2:** Characterization of donor age in groups I and II according to BMI.

	Group I [Donor Age <44.5 Years]	Group II [Donor Age >44.5 Years]
Group size [n]	58	59
Mean age ± SD [years]	34.95 ± 6.30	54.15 ± 7.89
Mean BMI ± SD [kg/m^2^]	28.19 ± 4.50	28.27 ± 4.82

**Table 3:** Characterization of the dataset based on BMI classification.

	Normal-Weight [BMI < 25]	Overweight [25 < BMI < 30]	Obesity [BMI > 30]
Group size [n]	32	47	35
Mean BMI ± SD [kg/m^2^]	23.22 ± 1.51	27.55 ± 1.33	33.95 ± 3.16
Mean age ± SD [years]	46.40 ± 13.3	44.13 ± 11.1	43.38 ± 12.4

**Table 4:** Analysis of the mean age and mean BMI classified by harvesting site.

	Thighs/Legs	Abdomen	Arm
Group size [n]	52	21	11
Mean age ± SD [years]	43.66 ± 13.13	45.76 ± 8.95	43.18 ± 13.17
Mean BMI ± SD [kg/m^2^]	27.97 ± 5.09	27.88 ± 3.45	31.54 ± 4.10

**Table 5:** Characterization of the flow cytometric dataset based on donor age (groups I and II) and BMI classification.

	Group I [Donor Age < 44.5 Years]	Group II [Donor Age > 44.5 Years]	
Group size [n]	10	9	
Mean age ± SD [years]	35.60 ± 6.08	58.67 ± 6.25	
Mean BMI ± SD [kg/m^2^]	27.14 ± 2.96	27.08 ± 3.90	
	**Normal-Weight** **[BMI < 25]**	**Overweight** **[25 < BMI < 30]**	**Obesity** **[BMI > 30]**
Group size [n]	6	8	5
Mean age ± SD [years]	46.17 ± 16.53	46.25 ± 9.08	47.40 ± 17.43
Mean BMI ± SD [kg/m^2^]	23.35 ± 1.58	27.42 ± 1.64	31.14 ± 0.71

**Table 6:** Flow cytometric analysis of cell surface markers of SVF cells: Assessment of relative proportions of cell populations within the SVF (n = 19).

	Cell Types	Mean ± SD	Variation Coefficient
CD34 vs. CD146	pericytes	25.78 ± 11.26	0.44
	adMSCs	9.13 ± 2.86	0.31
	endothelial cells	19.45 ± 8.31	0.43
CD144 vs. CD146	pericytes	26.6 ±10.49	0.39
	adMSCs	7.94 ± 2.78	0.35
	endothelial cells	14.47 ± 7.58	0.52
CD31 vs. CD146	pericytes	23.94 ± 9.15	0.42
	adMSCs	6.87 ± 3.09	0.45
	endothelial cells	21.69 ± 9.15	0.42

**Table 7:** Characterization of the cytokine dataset based on donor age (groups I and II) and BMI classification.

	Group I [Donor Age < 44.5 Years]	Group II [Donor Age > 44.5 Years]
Group size [n]	4	2
Mean age ± SD [years]	35.50 ± 4.04	62.50 ± 12.02
Mean BMI ± SD [kg/m^2^]	26.16 ± 3.24	24.96 ± 2.42
	**Normal-Weight** **[BMI < 25]**	**Overweight** **[25 < BMI < 30]**
Group size [n]	3	3
Mean age ± SD [years]	45.33 ± 22.37	43.67 ± 8.96
Mean BMI ± SD [kg/m^2^]	23.46 ± 1.26	28.05 ± 1.49

## Data Availability

The data presented in this study are available in this article and from the corresponding author.

## Supplementary Materials

The following supporting information can be downloaded at https://www.mdpi.com/article/10.3390/ijms27031351/s1.

## Appendix A

**Table A1:** Coordinates of the spots and a list of 36 different cytokines, chemokines, and acute-phase proteins on the Human Cytokine Array membrane.

Coordinate	Analyte/Control	Coordinate	Analyte/Control
A1, A2	Reference Spots	C7, C8	IL-4
A3, A4	CCL1/I-309	C9, C10	IL-5
A5, A6	CCL2/MCP-1	C11, C12	IL-6
A7, A8	MIP-1α	C13, C14	IL-8
A9, A10	CCL5/Rantes	C15, C16	IL-10
A11, A12	CD40 Ligand/TNFSF5	C17, C18	IL-12p70
A13, A14	Complement Component C/Ca	D3, D4	IL-13
A15, A16	CXCL/GROα	D5, D6	IL-16
A17, A18	CXCL/IP-10	D7, D8	IL-17A
A19, A20	Reference Spots	D9, D10	IL-17E
B3, B4	CXCL11/I-TAC	D11, D12	IL-18/IL-1F4
B5, B6	CXCL12/SDF-1	D13, D14	IL-21
B7, B8	G-CSF	D15, D16	IL-27
B9, B10	GM-CSF	D17, D18	IL-32α
B11, B12	ICAM-1/CD54	E1, E2	Reference Spots
B13, B14	IFN-gamma	E3, E4	MIF
B15, B16	IL-1α/IL-1F1	E5, E6	Serpin/PAI-1
B17, B18	IL-1β/IL-1F2	E7, E8	TNF-α
C3, C4	IL-1ra/IL-1F3	E9, E10	TREM-1
C5, C6	IL-2	E19, E20	Negative Control
