# Supplementary material for: Influence of Donor Age, Donor Body Mass Index, and Harvesting Site on Cell Preparations from Human Adipose Tissue
Source: Int J Mol Sci. 2026 Jan 29;27(3):1351. doi: 10.3390/ijms27031351 (PMC12897597; doi:10.3390/ijms27031351)
Supplement: Supplementary file 1 [file ijms-27-01351-s001.zip › Supplementary file.pdf]

Supplementary file

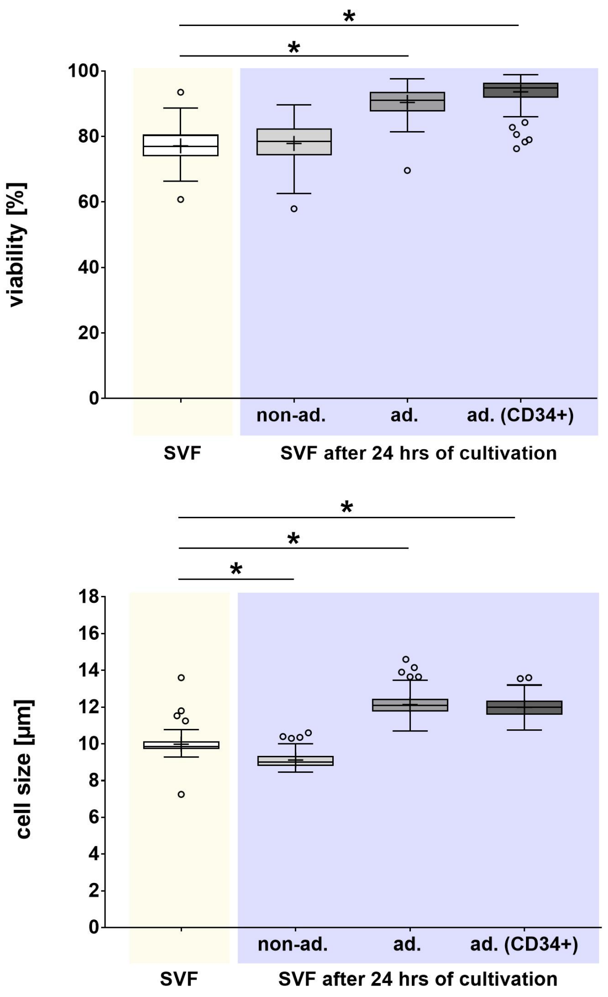

Figure S1. Quantification of the cell viability and cell size of SVF and SVF-derived cells. Cell viability and cell size of freshly isolated SVF and SVF-derived cells was presented as box plots, with medians, means (+), interquartile ranges and minimum/maximum values as whiskers (Tukey biweight). Data points below or above the whiskers are defined as outliers (circles). Shapiro-Wilk test indicated non-Gaussian distribution; statistical significance was calculated using the Kruskal-Wallis test with Dunn's multiple comparison post hoc test, *$p < 0.05$ significant compared to the SVF, n = 88 - 118).

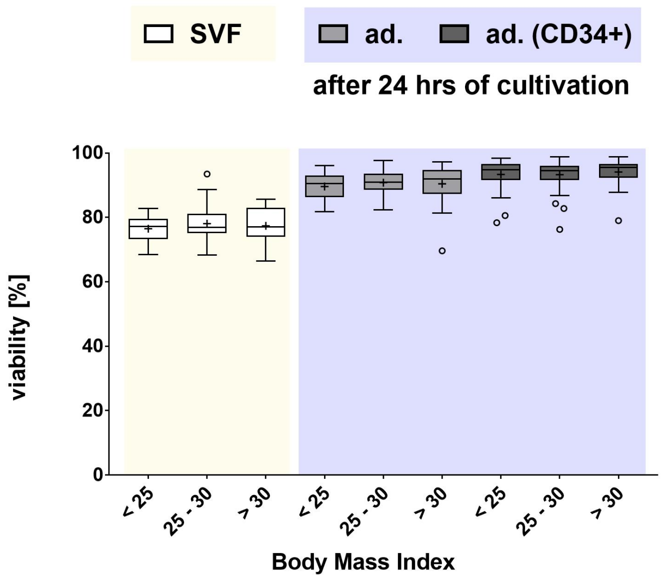

Figure S2. Comparative analysis of the effects of donor BMI on cell viability of SVF and SVF-derived cells. Cell viability of SVF and SVF-derived cells were presented as box plots, with medians, means (+), interquartile ranges, and minimum/maximum values as whiskers (Tukey biweight). Data points below or above the whiskers are defined as outliers (circles). For data sets indicating a non-Gaussian distribution (Shapiro-Wilk test), statistical significance was calculated using the Kruskal-Wallis test with Dunn's multiple comparison post hoc test. In contrast, data sets with a Gaussian distribution (Shapiro-Wilk test) were calculated using Ordinary One-Way ANOVA with Dunnett's multiple comparison post hoc test, $^*p < 0.05$ significance between normal-weight (BMI < 25), overweight (25 < BMI< 30) and obesity (BMI > 30), n = 106 - 113).

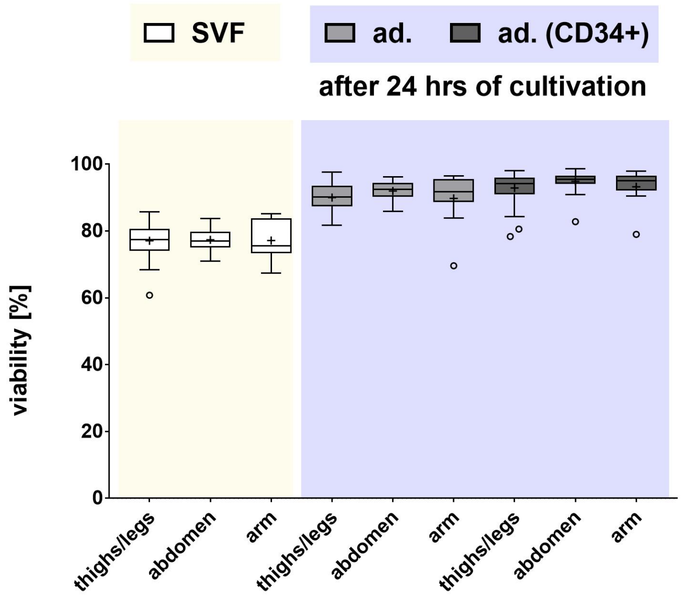

Figure S3. Comparative analysis of the effects of harvesting site on cell viability of SVF and SVF-derived cells. Cell viability of SVF and SVF-derived cells were presented as box plots, with medians, means (+), interquartile ranges, and minimum/maximum values as whiskers (Tukey biweight). Data points below or above the whiskers are defined as outliers (circles). For data sets indicating a non-

Gaussian distribution (Shapiro-Wilk test), statistical significance was calculated using the Kruskal-Wallis test with Dunn's multiple comparison post hoc test. In contrast, data sets with a Gaussian distribution (Shapiro-Wilk test) were calculated using Ordinary One-Way ANOVA with Dunnett's multiple comparison post hoc test, *$p < 0.05$, n = 76 - 82).
