# Supplementary figures and images for: Influence of Donor Age, Donor Body Mass Index, and Harvesting Site on Cell Preparations from Human Adipose Tissue
Source: Int J Mol Sci. 2026 Jan 29;27(3):1351. doi: 10.3390/ijms27031351 (PMC12897597; doi:10.3390/ijms27031351)

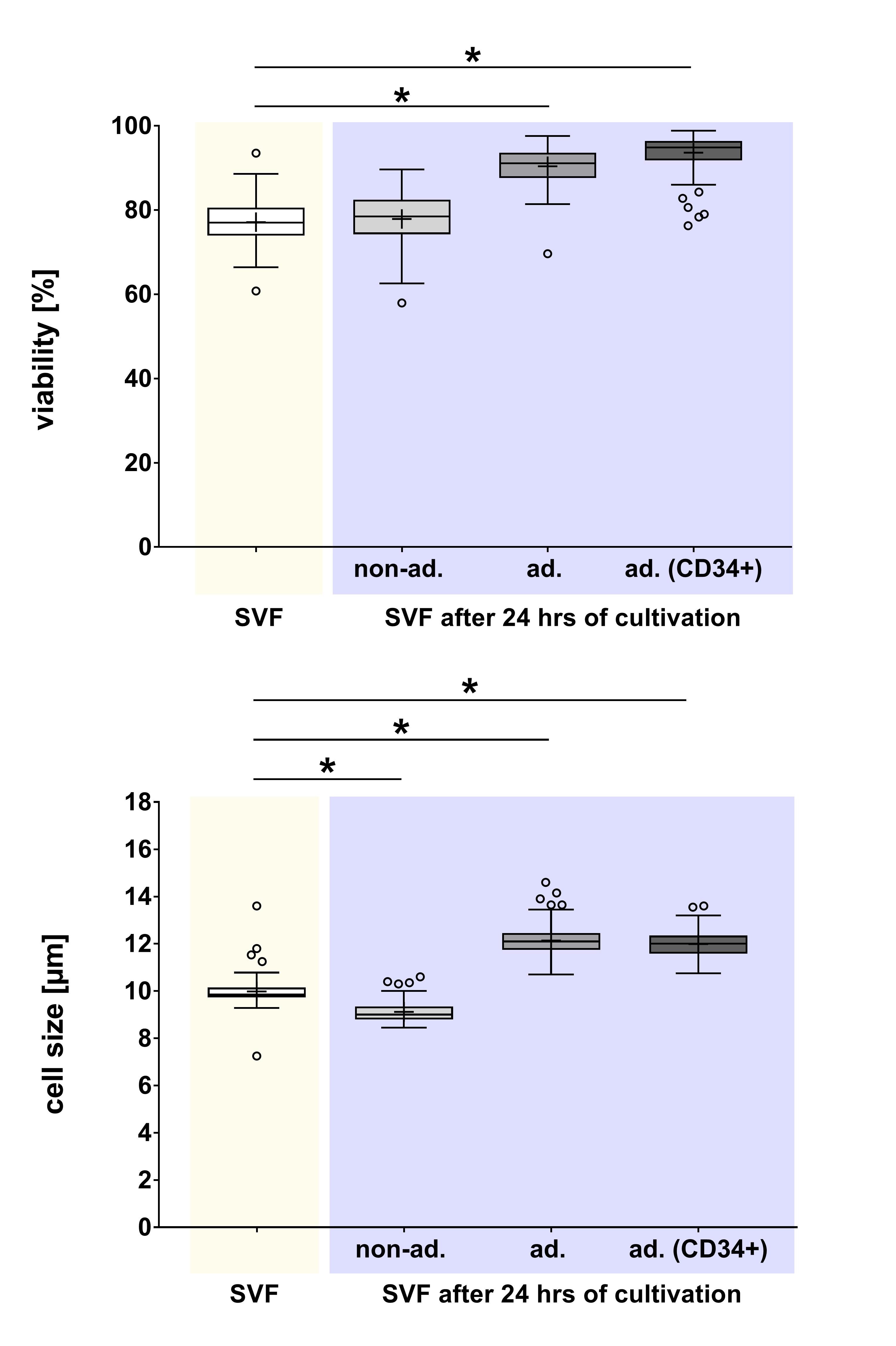

Supplement: Supplementary file 1 [file ijms-27-01351-s001.zip › Figure S1 Quantification of cell viability and cell size of SVF and SVF-derived cells.jpg]

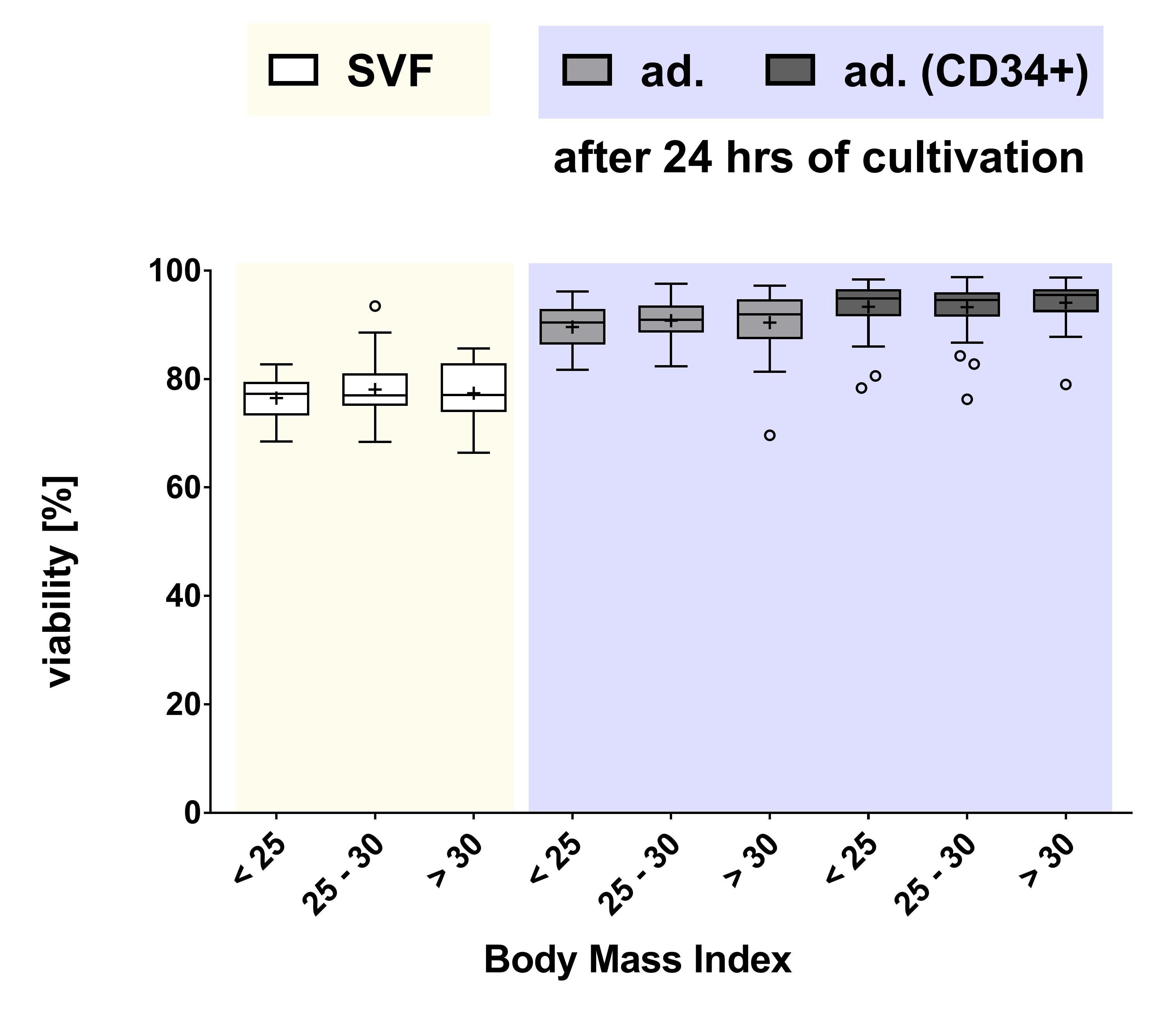

Supplement: Supplementary file 1 [file ijms-27-01351-s001.zip › Figure S2 Comparative analysis of the effects of donor BMI on cell viability of SVF and SVF-derived cells.jpg]

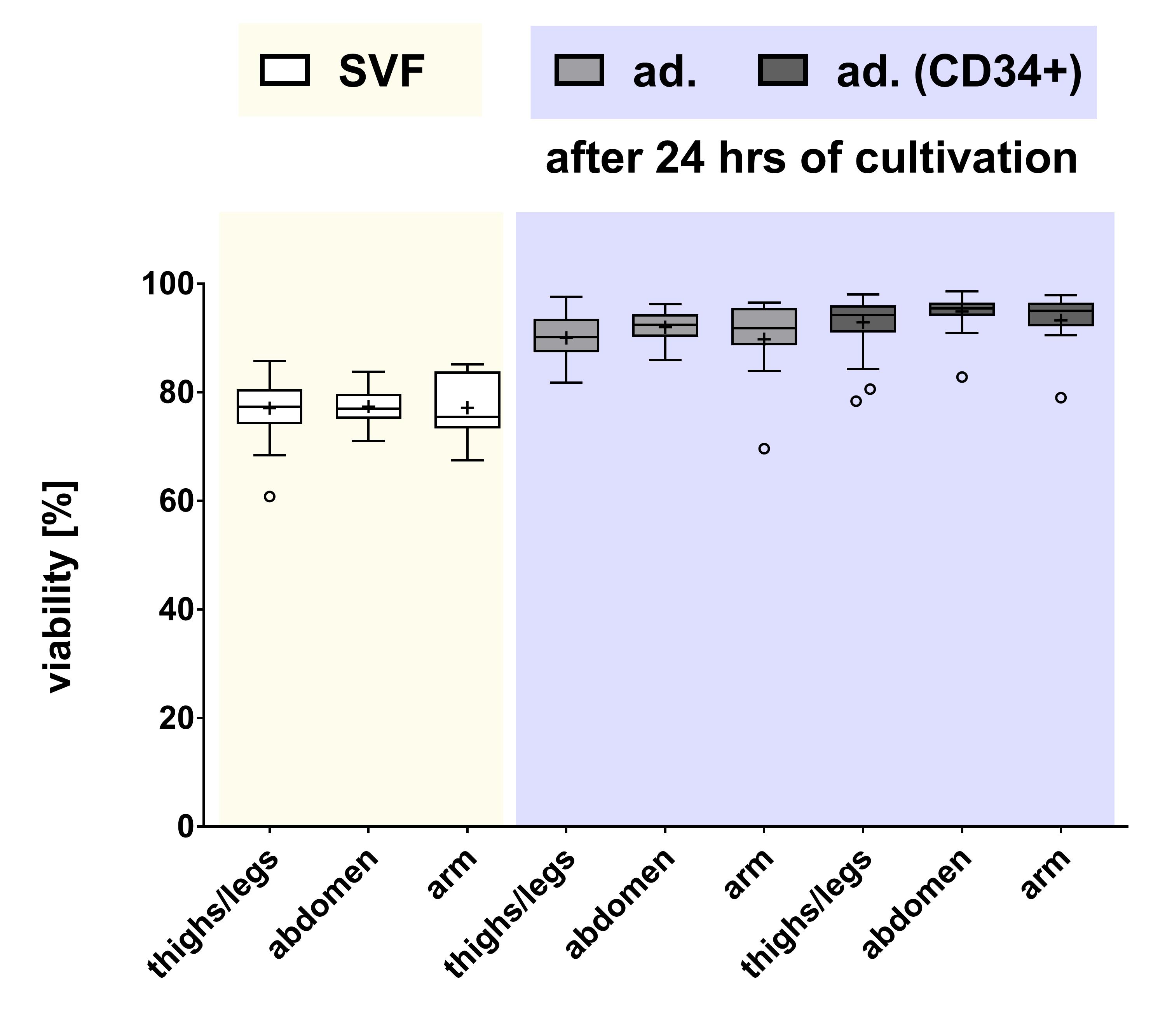

Supplement: Supplementary file 1 [file ijms-27-01351-s001.zip › Figure S3 Comparative analysis of the effects of harvesting site on cell viability of SVF and SVF-derived cells.jpg]
